# Regulatory T cells suppress virus-specific antibody responses to Friend retrovirus infection

**DOI:** 10.1371/journal.pone.0195402

**Published:** 2018-04-03

**Authors:** Tyler C. Moore, Ronald J. Messer, Kim J. Hasenkrug

**Affiliations:** Laboratory of Persistent Viral Diseases, Rocky Mountain Laboratories, NIAID, NIH, Hamilton, MT, United States of America; Wayne State University School of Medicine, UNITED STATES

## Abstract

Recent vaccine studies with experimental antigens have shown that regulatory T cells (Tregs) constrain the magnitude of B cell responses. This homeostatic Treg-mediated suppression is thought to reduce the potential of germinal center (GC) responses to generate autoreactive antibodies. However, essentially opposite results were observed in live influenza infections where Tregs promoted B cell and antibody responses. Thus, it remains unclear whether Tregs dampen or enhance B cell responses, especially during live viral infections. Here, we use mice infected with Friend retrovirus (FV), which induces a robust expansion of Tregs. Depletion of Tregs led to elevated activation, proliferation, and class switching of B cells. In addition, Treg depletion enhanced the production of virus-specific and virus-neutralizing antibodies and reduced FV viremia. Thus, in contrast to influenza infection, Tregs either directly or indirectly suppress B cells during mouse retroviral infection indicating that the ultimate effect of Tregs on B cell responses is specific to the particular infectious agent.

## Introduction

Regulatory T cells (Tregs) are immunosuppressive CD4^+^ T cells that express the transcription factor Foxp3 and play a predominant role in immunological homeostasis and the prevention of autoimmune diseases [1]. Tregs can also dampen immune responses to infectious agents (reviewed in [2]). Many studies have focused on effector T cells as targets of Treg suppression, but recent evidence shows that B cells and germinal center responses also fall under the control of Tregs [3–5] as a mechanism to prevent the production of autoantibodies [6–8].

Treg depletion studies have revealed a role for Tregs in preventing an outgrowth of non-antigen specific B cells in germinal centers [4]. Further evidence for Treg suppression of B cells has been shown in recent immunization studies employing the experimental antigen NP-ovalbumin [9, 10]. In contrast to studies done using experimental antigens such as NP-KLH, Ova or sheep red blood cells, which showed Treg-mediated suppression of B cell/antibody responses, a study done in mice infected with live influenza virus showed that depletion of Tregs severely reduced, rather than enhanced, B cell responses and antibody production [11]. These studies suggested context dictates whether Tregs enhance or suppress the production of antibodies. They also illustrated that while studies using model antigens are very important for elucidating basic mechanisms of immunological responses, it is also essential to study live viral infections, which induce much more complex responses and may give surprising results. In this regard, we sought to determine the effect of Tregs on antibody responses to a mouse retroviral infection.

In the current study we used mice infected with Friend virus (FV), a naturally occurring mouse retrovirus that causes acute infections that become chronic [12, 13]. FV infections induce the activation and proliferation of natural or thymus-derived tTregs, but does not induce the conversion of conventional T cells into Tregs [14]. FV-induced Tregs have previously been demonstrated to suppress the function of both CD4+ [15] and CD8+ T cells [16, 17]. FV infections were done in B6.FOXP3-DTR mice [18], which express the human diphtheria toxin (DT) receptor downstream and under transcriptional control of the FOXP3 locus. FOXP3 is a transcriptional factor that is required for Treg differentiation and function [19]. Injection of DT into these mice specifically depletes Tregs [18]. A role for Tregs in suppressing antiviral immune responses was originally shown in studies using the FV model [17], but until now Treg-mediated effects have focused on T cells [15, 20–22]. Treg-mediated influences on FV-specific antibody responses have not yet been investigated. The current results demonstrate potent suppression by Tregs on the development of specific antibody responses to acute retroviral infection.

## Materials and methods

### Mice

Experiments were conducted using female B6.129(Cg)-*Foxp3tm3(DTR/GFP)Ayr*/J (B6.FOXP3-DTR-GFP) mice (obtained from Jackson Labs and bred at the Rocky Mountain Laboratories, Hamilton, MT) [18]. All of the mice were 10–20 weeks old at the beginning of the experiments.

### Ethics statement

Rocky Mountain Laboratories is fully accredited by AAALAC (2017). All mice used in these experiments and were treated in accordance with an RML/NIAID/NIH Animal Care and Use Committee-approved protocol and the regulations and guidelines of the National Institutes of Health. Isoflurane inhalation anesthesia was used for procedures and overdose of isoflurane inhalation anesthesia was used for euthanasia.

### Virus, diphtheria toxin, and injections

The FV stock used in these experiments was an FV complex containing replication competent B-tropic Friend murine leukemia helper retrovirus and replication defective polycythemia-inducing spleen focus-forming retrovirus (free of lactate dehydrogenase–elevating virus) [23]. Mice were infected by i.v. injection of 20,000 spleen focus forming units (SFFU) in 0.2 mL via the retroorbital sinus. Tregs were depleted by injecting mice i.p. with 0.5 ug of DT (Sigma) on days 0, 3, and 6 days post infection. Mice were bled by capillary tube through the retroorbital sinus on days 7, 14, and 21 post infection. Plasma was saved for antibody and viremia assays, and cells were analyzed by flow cytometry following red blood cell lysis with ammonium chloride-potassium buffer (ACK) for 5 minutes. Spleens were harvested at 14 and 21 days post infection, homogenized, processed, and stained for flow cytometric analysis following red blood cell lysis with ACK for 5 minutes. An aliquot of homogenized spleen cells was used for the infectious center assay prior to red blood cell lysis.

### Surface and intracellular antibody staining and flow cytometry

The following antibodies were used for cell surface staining: anti-CD4 (RM4-5, BioLegend), anti-CD19 (1D3, BioLegend), anti-CD38 (90, eBio), anti-CD138 (281–2, BD), anti-CD86 (Gl1, Biolegend), anti-Ter119 (TER-119, eBio), anti-GL7 (GL-7, eBio), anti-mouse IgD (11–26, eBio) anti-mouse IgM (11/41, eBio), anti-PD1 (J43, eBio), anti-CD43 (1B11, BioLegend), CD62L (MEL-14, BioLegend). Following surface staining, intracellular staining was performed with the FOXP3 permeabilization and fixation kit from eBiosciences according to the manufacturers recommendations using the following antibodies: Ki-67 (SolA15, BD) or FOXP3 (FJK-16s, eBio). FOXP3 expression in cells from FOXP3-DTR mice was also analyzed by GFP expression. Lymphocyte populations were initially gated on the basis of forward scatter versus side scatter. B cells were gated as CD19^+^CD4- cells within the lymphocyte gate. GC B cells were gated as CD38^-^GL7^+^ cells within the B cell gate and plasmablasts were gated as CD38^+^CD138^+^ cells within the B cell gate. CD4 T cells were gated as CD4^+^ cells within the lymphocyte gate. See S1 Fig for gating strategies. Flow cytometic data were collected with an LSRII (BD Biosciences) and analyzed using FlowJo software (Tree Star).

### IgG ELISA

ELISA plates were coated with 10μg FV-B/well overnight in 100μl coating buffer (eBiosciences). Excess antigen was washed 3 times with PBS-Tween. Plates were blocked with PBS + 5% FBS for 2 hours at room temperature, then washed twice with PBS-Tween. Plasma diluted in PBS-Tween + 5% FBS was added at 100μl/well and incubated for 3 hours at room temperature or overnight at 4 deg C. After 4 washes with PBS-Tween, the plates were incubated with a 1:1000 dilution (in PBS-Tween + 5% FBS) goat anti-mouse IgG-HRP secondary (eBiosciences). After 4 washes with PBS-Tween, the plates were developed with TMB substrate (5% 2M NaOAc + 4% TMB + 0.05% H_2_O_2_ in H_2_O) for 15 minutes and then stopped with 2M H_2_SO_4_. OD450 values were recorded, and μg/mL plasma was calculated by using serial dilutions of purified quantified mAb48, a FV-specific IgG2a, as a standard [24]. The limit of detection was approximately 77 ng FV-binding IgG/mL plasma.

### Neutralizing antibody, Infectious-Center (IC), and viremia assays

Plasma samples were assayed for neutralizing antibodies as described previously, by analyzing FV infectivity of susceptible *Mus dunni* cells following incubation with dilutions of plasma [23, 25]. The titer was defined as the dilution at which >50% of the input virus was neutralized. The IC assays were performed as described previously by seeding dilutions of splenocyte suspensions onto susceptible *Mus dunni* cells [23, 25]. For viremia assays, plasma samples frozen at -80°C were thawed once and titrated using focal infectivity assays on susceptible *Mus dunni* cells pretreated with 4 μg/mL Polybrene as described [26]. The cultures were incubated for 2 days, fixed with ethanol, and labeled first with F-MuLV-envelope-specific mAb 720 [27] and then with goat anti-mouse HRP (eBiosciences) followed by 3-amino-9-ethylcarbazole (Sigma) as a substrate to detect foci.

## Results

**Fig 1:**
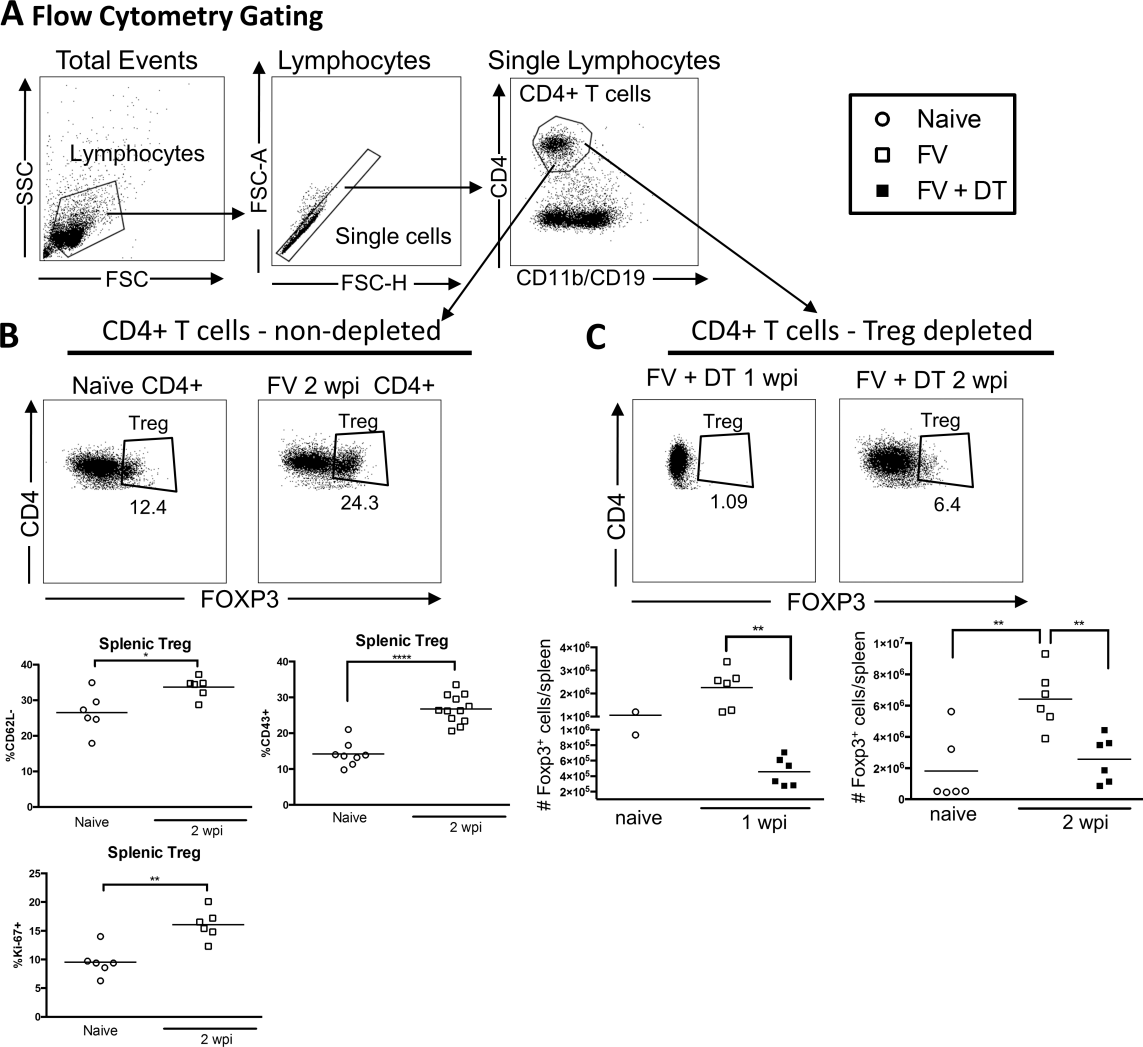
Depletion of Tregs during FV infection. Gating strategy for Tregs **(A-C)**. B6-FOXP3-DTR mice were injected with 20,000 SFFU i.v. FV and left untreated (**B**) or injected with DT on day 0, 3, and 6 relative to infection (**C**). Spleens were harvested at 1 (**C**) or 2 wpi (**B, C**) and cells were analyzed by flow cytometry compared to naïve mice. (**B**) Splenic Tregs from non-depleted mice were analyzed for CD43, CD62L and Ki-67 expression, represented as a percentage of Tregs. (**C**) The total number of Tregs at 1 wpi or 2 wpi with or without Treg depletion was compared to the levels in naïve mice. Each dot represents an individual mouse pooled from experiments with 2–6 mice per group. Lines indicate the mean of each group. * P < .05, **P < .01, ****P < .0001 as determined by ordinary one-way ANOVA with Tukey post-test for multiple comparisons or an unpaired two-tailed t test for comparisons between only two groups.

In order to investigate the effects of Tregs on B cell responses we depleted Tregs *in vivo* with DT injections on days 0, 3, and 6 relative to infection. Confirming previous studies [14, 28, 29], we found a significant expansion and activation of Tregs at 2 weeks post infection with FV in non-depleted mice (Fig 1A and 1B). In DT-treated mice, there was significant depletion of Tregs at 1wpi (Fig 1C), and as previously reported [9], Tregs rebounded to approximately naive levels by one week later (Fig 1C, 2wpi). However, the depletion of Tregs during acute infection negated the FV-induced expansion of the Treg subset (Fig 1C).

**Fig 2:**
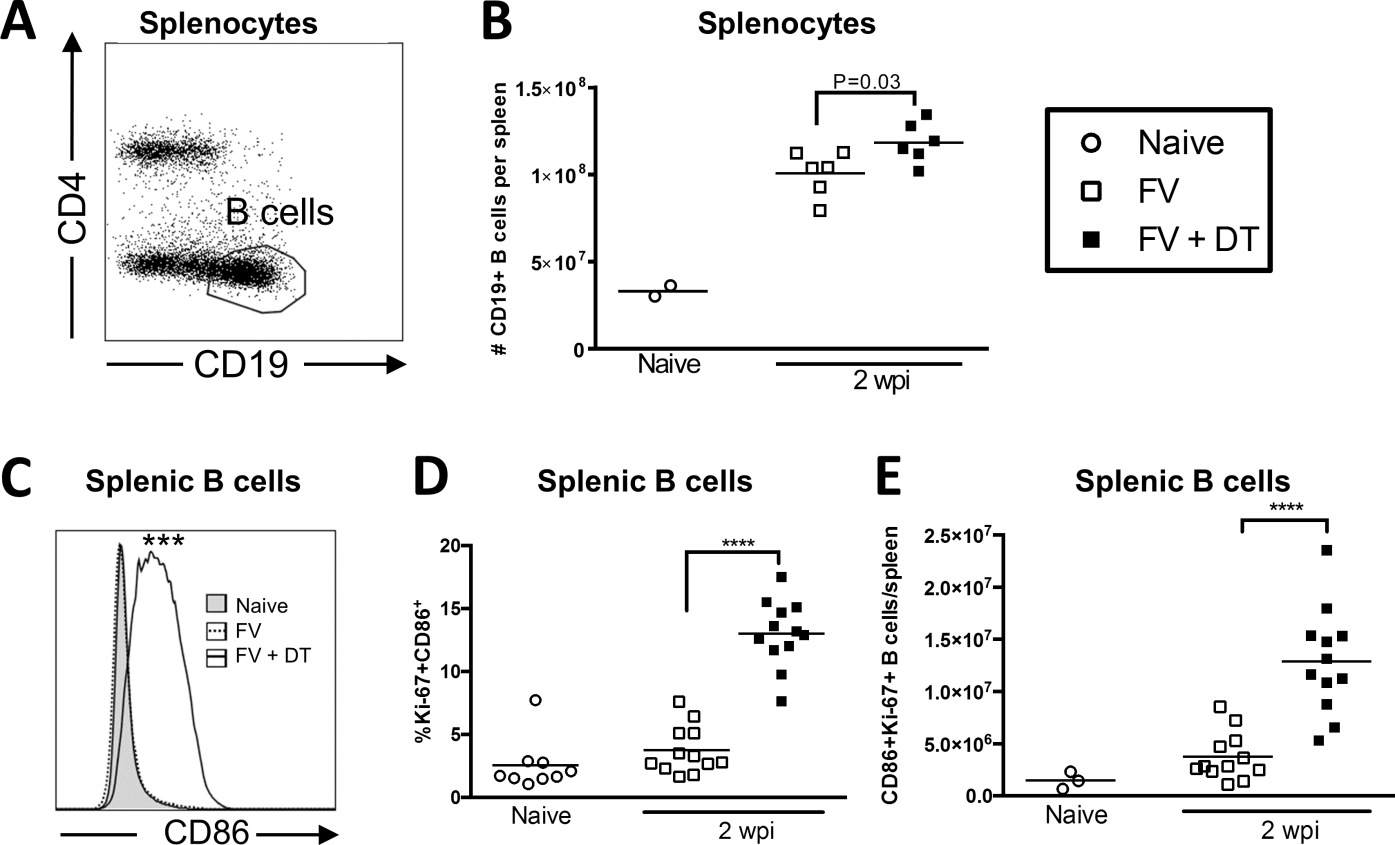
Transient Treg depletion enhances B cell activation in FV-infected mice. B6.FOXP3-DTR mice were injected with 20,000 SFFU i.v. and left untreated or injected with 0.5 μg DT i.p. at 0, 3, and 6 dpi. Spleens were harvested at 2 wpi and total splenocytes (**A, B**) or CD19^+^CD4^-^ B cells (**C, D, E**) were analyzed by flow cytometry. **(B)** Absolute numbers of CD19^+^ cells per spleen. (**C**) Representative histograms of CD86 expression on B cells; Mean MFI of Naïve: 221, FV: 304.8, FV + DT: 506.5, P < .001. (**D**) %Ki-67^+^CD86^+^ B cells in the spleen. **(E)** Number of Ki-67^+^ CD86^+^ B cells/spleen. Each dot in (**B, C, D, E)** represents an individual mouse pooled from experiments with 2–6 mice per group. Lines indicate the mean of each group. *P < .05, **P < .01, ***P < .001, ****P < .0001 as determined by ordinary one-way ANOVA with Tukey post-test for multiple comparisons.

We next sought to determine the effect of Treg depletion on B cell activation during FV infection. Treg depletion was associated with a significant increase in overall B cell (CD19^+^ CD4^-^) numbers in the spleen (Fig 2A and 2B) and significantly elevated activation as indicated by increased CD86 expression (Fig 2C). In addition, the activated B cells were proliferating as indicated by CD86 and Ki-67 co-expression (Fig 2D) and increased absolute numbers of CD86, Ki67 double positive cells (Fig 2E).

**Fig 3:**
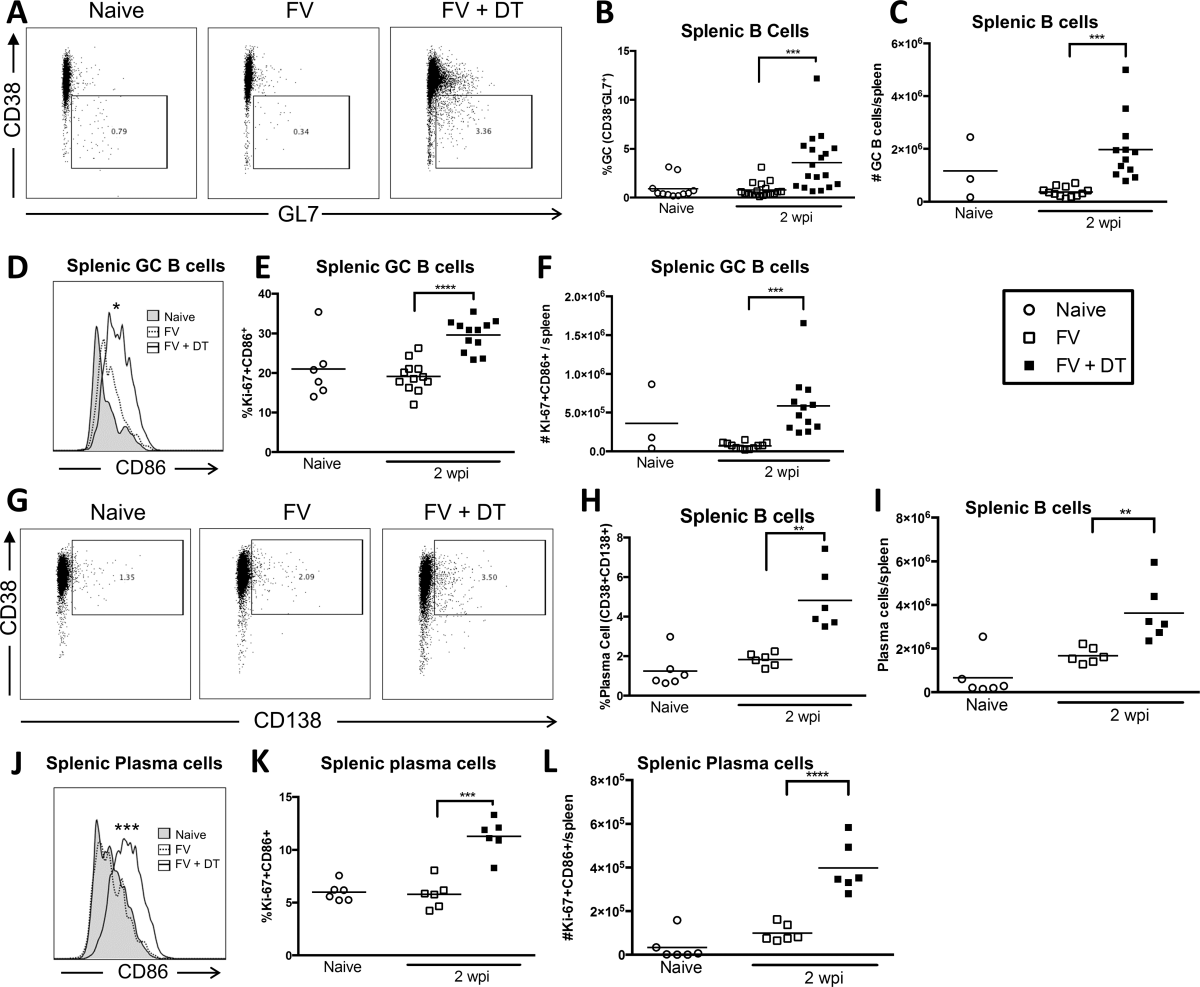
Transient Treg depletion enhances GC B cell and plasma cell responses in FV-infected mice. B6.FOXP3-DTR mice were injected with 20,000 SFFU i.v. and left untreated or injected with 0.5 μg DT i.p. at 0, 3, and 6 dpi. Spleens were harvested at 2wpi and CD19^+^CD4^-^ B cells (**A–C, G–I**), CD38^-^Gl7^+^ germinal center B cells (**D–F**), or CD38^+^CD138^+^ plasma cells (**J–L**) were analyzed by flow cytometry. Representative FACS plots showing GC B cell **(A)** or plasma cell gates **(G)**. **(D, J)** Representative histograms of CD86 expression on GC B cells; (**D**) Mean MFI of Naïve: 611, No DT: 800.0, DT: 944.3, P < .05, or plasma cells (**J**) Mean MFI of Naïve: 476.5, No DT: 542.2, DT: 1340.7, P < .001. Quantification of %Ki-67^+^CD86^+^ (**E, K)**, and total number of Ki-67^+^CD86^+^ **(F, L)** GC B cells or plasma cells as indicated. Each dot represents an individual mouse pooled from experiments with 2–6 mice per group. Lines indicate the mean of each group. * P < .05, **P < .01, ***P < .001, ****P < .0001 as determined by ordinary one-way ANOVA with Tukey post-test for multiple comparisons.

The effect of Tregs on the maturation of B cells during FV infection was examined by analyzing germinal center (GC) and plasma cells. The gating strategies for these subsets are shown in Part A of S1 Fig and Fig 3A and 3G). We did not detect a significant increase in either splenic GC B cells (Fig 3B and 3C) or plasma cells (Fig 3H and 3I) at 2 wpi with FV compared to naïve mice, but Treg depletion significantly increased both the proportion and absolute numbers of both subsets. Furthermore, the activation (Fig 3D and 3E) and proliferation (Fig 3E and 3F) of GC B cells and plasma cells (Fig 3J–3L) were significantly increased. Thus, Treg depletion strongly enhanced the activation and expansion of differentiated B cells during acute FV infection.

**Fig 4:**
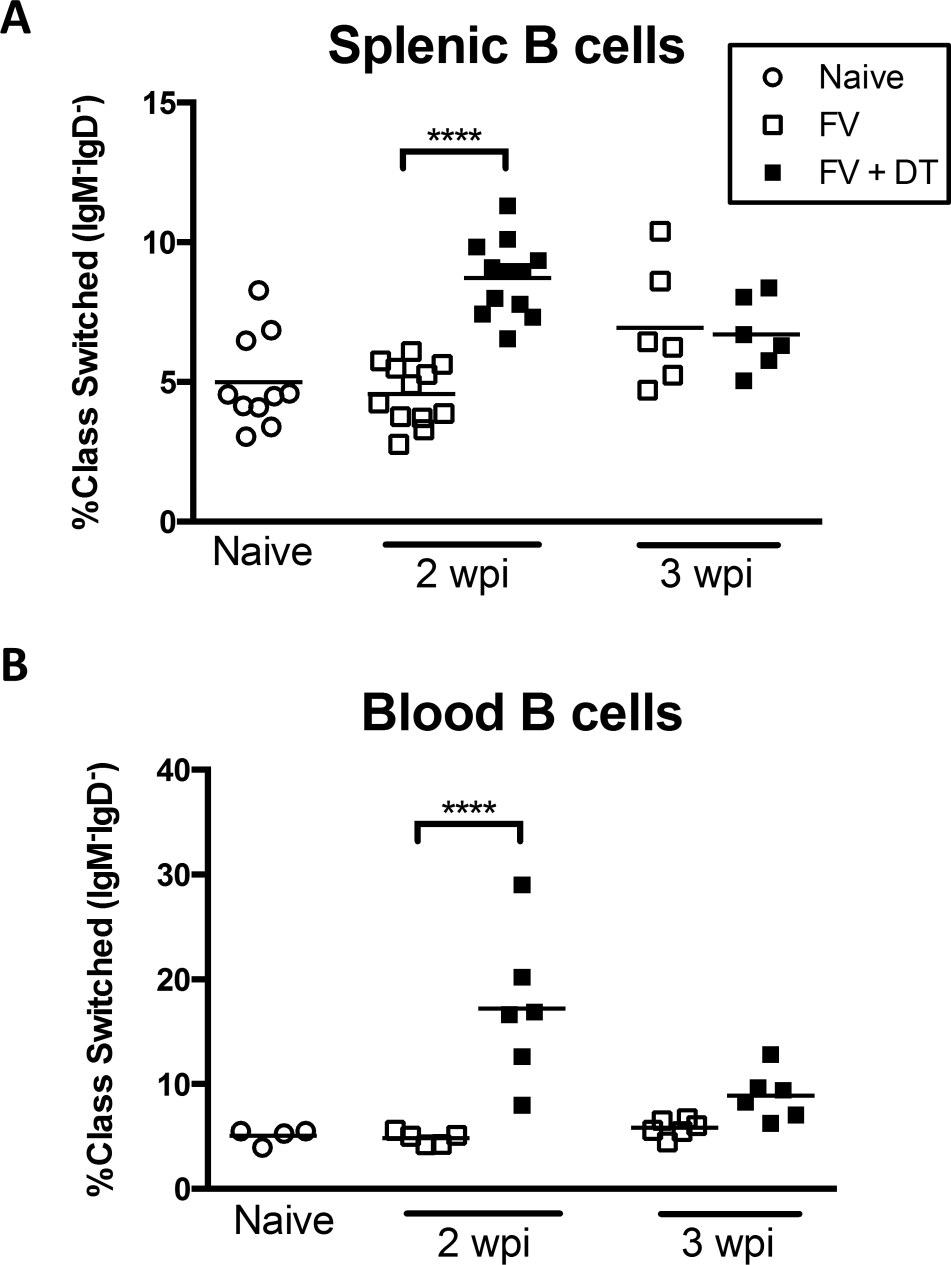
Transient Treg depletion enhances B cell class switching in FV-infected mice. B6.FOXP3-DTR mice were injected with 20,000 SFFU i.v. and left untreated or injected with 0.5 μg DT i.p. at 0, 3, and 6 dpi. Mice were bled and spleens were harvested at 2wpi or 3 wpi and total CD19^+^CD4^-^ B cells were analyzed for IgM and IgD expression by flow cytometry. Each dot represents an individual mouse pooled from experiments with 2–6 mice per group. Lines indicate the mean of each group. ****P < .0001 as determined by ordinary one-way ANOVA with Tukey post-test for multiple comparisons.

An important aspect of the immune response to FV is T-dependent class switching of the antibody response [30]. Because we observed enhanced activation of B cells in Treg depleted mice during FV infection it was of interest to determine if there was also enhanced class switching. Indeed, a significantly higher proportion of IgM^-^IgD^-^ class-switched B cells in both the spleen (Fig 4A) and the blood (Fig 4B) of Treg-depleted mice were observed at 2 wpi. This difference essentially normalized by 3 weeks post infection. These results indicated that FV-induced Tregs suppressed B cell class switching early in the infection.

**Fig 5:**
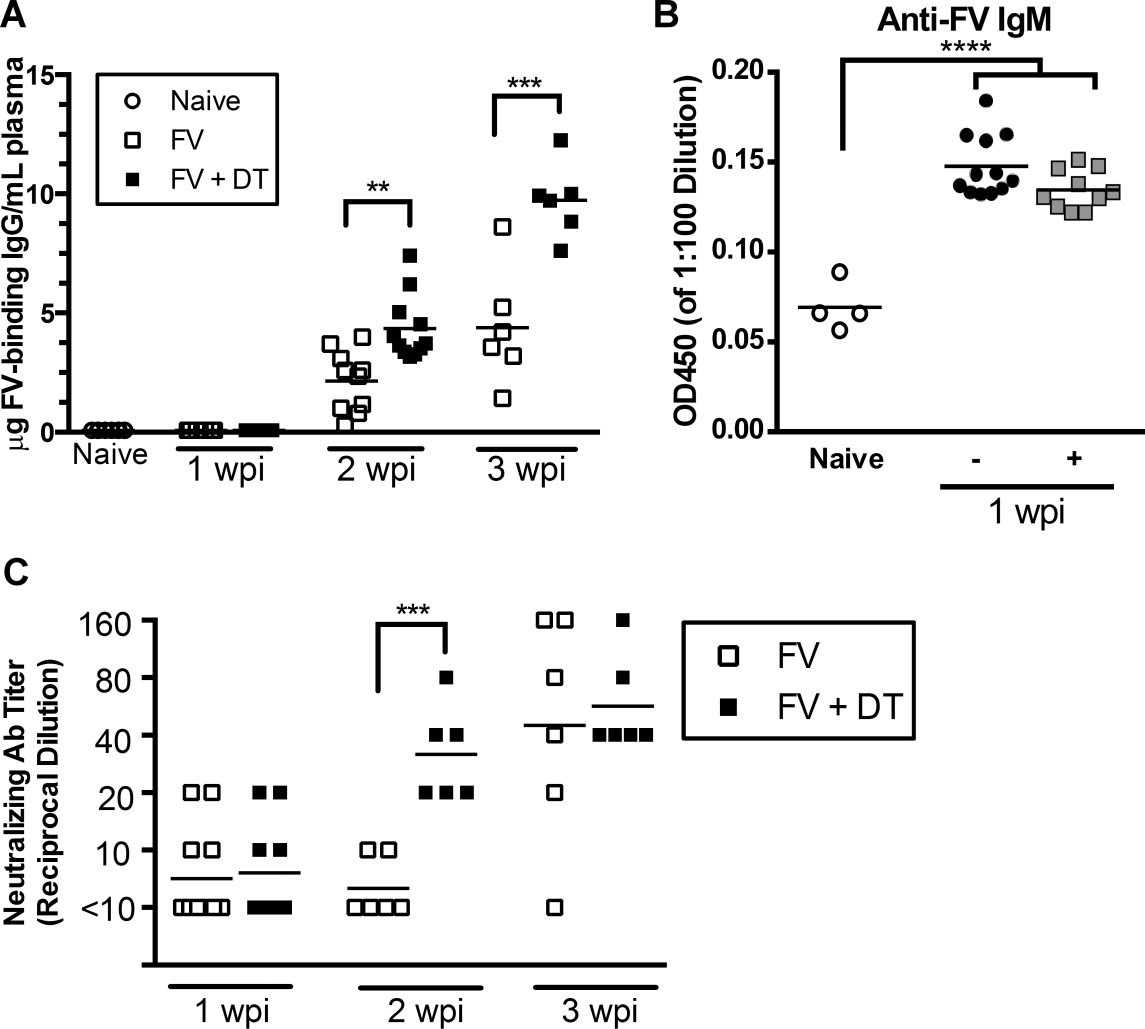
Transient Treg depletion enhances antibody responses against FV. B6.FOXP3-DTR mice were injected with 20,000 SFFU i.v. and left untreated or injected with 0.5 μg DT i.p. at 0, 3, and 6 dpi. Mice were bled at 1, 2, and 3 wpi and plasma was analyzed for FV-binding IgG by ELISA (**A**), IgM (1:10 dilution) by ELISA (**B**), or FV-neutralizing antibody by neutralizing antibody assay (**C**). Each dot represents plasma from an individual mouse. Lines indicate the mean of each group. ***P < .001, as determined by ordinary one-way ANOVA with Tukey post-test for multiple comparisons.

Given the suppressive effect of Tregs on B cell activation and class switching, it was of interest to determine whether lack of Treg control in depleted mice resulted in higher titers of virus-specific antibodies. An ELISA was used to measure levels of FV-binding IgG in plasma from the Treg-depleted and control animals. It is important to note that this assay only detects free antibodies in the plasma, and any antibody complexed with virus would not be detected. No FV-binding IgG was detectable in naïve mice or FV infected mice at 7 dpi (limit of detection 80ng/mL), although IgM-binding antibodies were detectable (Fig 5B). Treg depletion did not affect the levels of IgM subclass antibodies at 1 wpi. By 2wpi, all FV-infected mice had detectable FV-binding IgG titers but the titers were significantly higher in Treg-depleted mice (Fig 5A). By 3wpi, the virus-specific IgG titers in the Treg-depleted animals were approximately double those of non-depleted controls (Fig 5A). Neutralizing antibodies play a particularly important role in FV infection [26], so it was important to measure virus-neutralizing antibodies as well as virus-binding IgG. By 2wpi the neutralizing antibody titers in Treg-depeleted mice were significantly higher than in untreated mice (Fig 5C). Interestingly, by 3wpi, the neutralizing antibody titers of most of the non-depleted control animals had caught up with the Treg-depeleted mice (Fig 5C). Together, these results indicated that Tregs slowed the development and magnitude of FV-specific antibody responses during the acute phase of infection.

**Fig 6:**
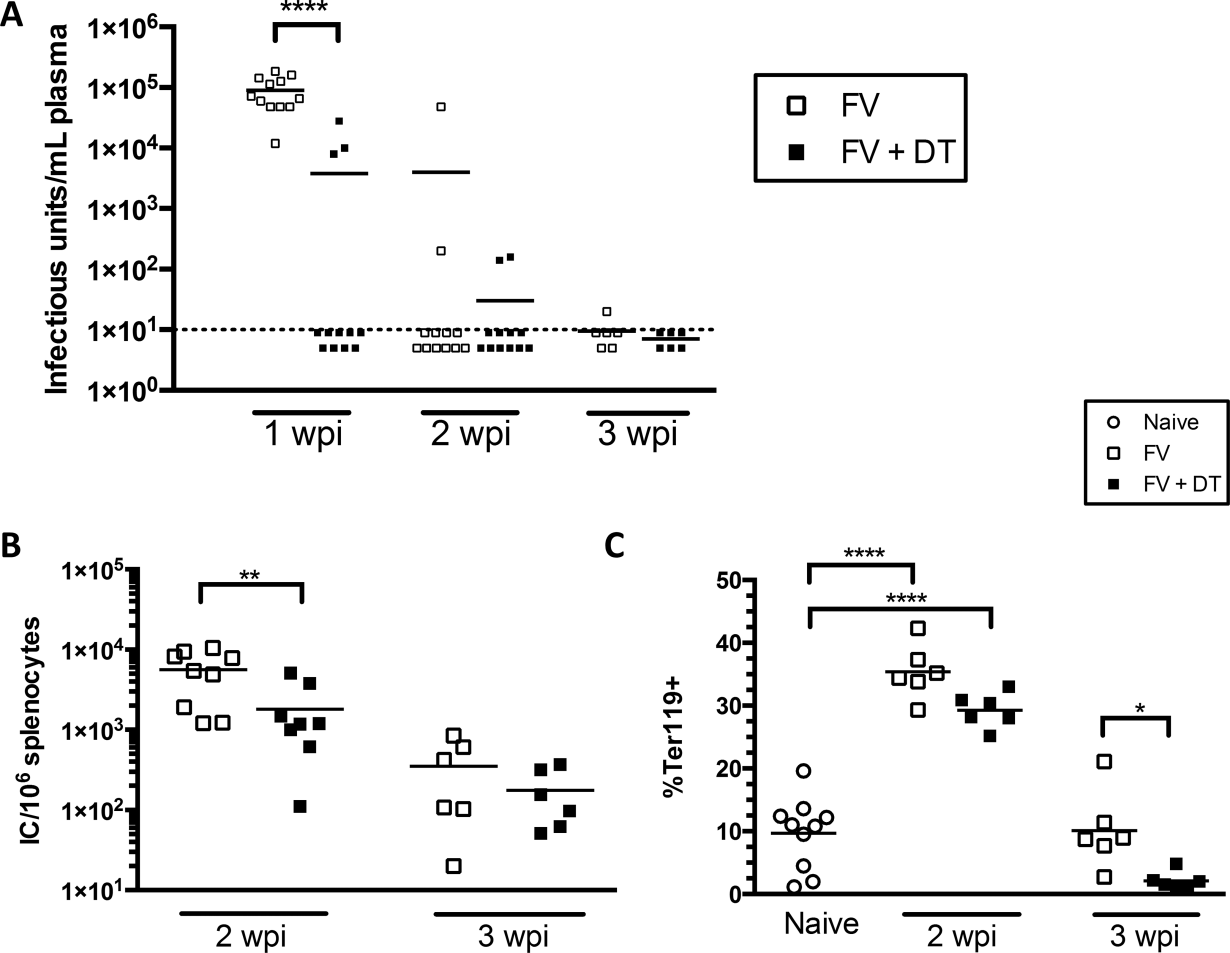
Transient Treg depletion enhances antibody responses against FV. B6.FOXP3-DTR mice were injected with 20,000 SFFU i.v. and left untreated or injected with 0.5 μg DT i.p. at 0, 3, and 6 dpi. Mice were bled at 1, 2, and 3 wpi and infectious units/mL blood were determined by viremia assay (**A**). Spleens were harvested at 2 and 3 wpi (**B, C**). Infectious centers per spleen were determined by infectious center assay and %Ter119^+^ cells were analyzed by flow cytometry. Each dot represents an individual mouse from experiments with 2–6 mice per group. Lines indicate the mean of each group. * P < .05, **P < .01, ****P < .0001, as determined by ordinary one-way ANOVA with Tukey post-test for multiple comparisons.

An essential role for neutralizing antibodies in FV infections is the control of plasma viremia [26, 31, 32] and that neutralizing antibody response has been shown to be T cell-dependent [30]. Because we observed higher neutralizing antibody titers in Treg-depleted mice during FV infection (Fig 5C), it was of interest to determine whether Treg depletion impacted control of plasma viremia. Compared to controls, which all had very high viremia at 1wpi, viremia was detectable in only 3 out of 12 Treg-depleted mice at 1 wpi (Fig 6A). By 2 wpi, viremia was reduced to undetectable levels in most of the mice of both Treg-depleted and non-depleted groups (Fig 6A). Despite the lack of plasma viremia in most Treg-depleted mice at 2 wpi, the level of infected cells (as detected by infectious center assay, Fig 6B), which is primarily controlled by CD8^+^ T cells [33, 34], remained elevated in both groups, albeit at reduced levels in Treg-depleted mice. Essentially the same result was obtained by measuring the levels of Ter119^+^ erythroid precursor cells, (Fig 6C), which proliferate in response to erythropoietin receptor stimulation from the viral protein, gp55 [35]. The reduced levels of infected cells in Treg-depleted mice compared to controls most likely reflect Treg-mediated control of the CD8^+^ T cell response, as previously described [15, 29, 36], but may also have been impacted by better antibody responses.

## Discussion

The current study shows that Tregs suppress antigen-specific B cell responses during acute FV infection of mice. We were able to demonstrate that transient depletion of total Tregs during FV infection significantly enhanced the kinetics of activation, proliferation, and class switching of splenic B cells (Figs 2–4). These effects were associated with production of FV-binding IgG that developed with faster kinetics than in non-depleted mice (Fig 5A). No effects on the production of IgM was observed suggesting that the mechanism of B cell suppression may be indirect via the suppression of CD4+ T cell help. Treg-mediated suppression of CD4+ T cells has previously been observed [15]. Since Tregs were rebounding by 2 wpi (Fig 1C) the results also suggest that early suppression during the induction phase of the immune responses has a long-term effect on the immune responses. Such long-term effects by transient Treg depletion on CD8+ T cell responses has previously been demonstrated [20]. Furthermore, we also observed the production of FV-neutralizing antibodies with faster kinetics (Fig 5C) and this was associated with more rapid control of viremia (Fig 6A). Although control of viremia in Treg-depleted mice preceded a detectable difference in virus-binding or virus-neutralizing antibodies compared to non-depleted mice (Figs 5 and 6), this is likely because the ELISA and neutralizing antibody assays can only detect free antibodies and not antibodies already bound to virus. Thus, Treg-depleted mice likely had elevated FV-specific antibody responses already at 7 dpi, but they were bound to virus, consistent with observed reductions in viremia (Fig 6). It was interesting that non-Treg-depleted mice had detectable antibody titers at 2 wpi (Fig 5) but not detectable GC or plasma B cells (Fig 3). This likely reflects the fact that the FV infection in B6 mice is limited (less than 10^4^ infectious centers per spleen at the peak of infection, Fig 6) and so is the B cell response. Although increases in GC B cells were not detected in non-depleted mice at 2 wpi while antibodies were detectable, it must be considered that each B cell is capable of producing thousands of antibodies per second [37]. Thus, the signal for detection of antibody is amplified by orders of magnitude compared to the cellular response.

These findings reveal that in addition to Treg-mediated suppression of CD4^+^ and CD8^+^ T cells, B cell responses are also either direct or indirect targets of Treg-mediated suppression during FV infection. Recent studies of vaccine responses to model antigens found that Tregs suppress GC responses through CTLA-4-mediated downregulation of CD86 on the surface of B cells [9, 10]. The results herein show that FV-induced Tregs significantly suppressed B cell expression of CD86, differentiation into GC B cells and plasma cells (Figs 2 and 3), and also suppressed B cell class switching (Fig 4). The interaction between CD86 on the surface of B cells and CD28 on the surface of CD4^+^ T cells is essential for the induction and maintenance of T follicular helper (Tfh) cells within the GCs, as well as the maturation of B cells into both GC B cells and antibody-secreting plasma cells [9, 38]. These results support the notion that Tregs control antiviral antibody responses by downregulating B cell CD86 expression, which reduces costimulation of Tfh cells. It has also been shown *in vitro* that Tregs can directly suppress the antibody production of mature B cells in the absence of Tfh cells [39]. Collectively, these results suggest that Tregs induced during FV infection could be suppressing B cell activation and virus specific antibody production through multiple mechanisms.

It is interesting that despite robust expansion and activation of conventional Tregs during a normal FV infection (Fig 1), the mice are nonetheless capable of mounting a B cell response and producing virus-binding and neutralizing antibodies, albeit to a lesser magnitude and with delayed kinetics compared to Treg-depleted mice. By 3 wpi, antibody-mediated clearance of viremia in normal mice was essentially as good as in Treg-depleted mice (Fig 6A). Thus, B cells are able to overcome Treg suppression to mount fully effective humoral immunity. In contrast, CD8^+^ T cells, which control cellular infection levels, become increasingly dysfunctional during FV infection because of Treg-mediated suppression [20], and chronically infected cells persist indefinitely [12, 13]. The long-lasting suppression of CD8^+^ T cells may be necessary because of their ability to cause immunopathological damage through mechanisms such as release of cytotoxic granules and secretion of cytokines like TNFα. These experiments highlight the elegant control of the immune system in balancing antiviral immunity with control over autoimmune responses. While FV infection isn’t completely cleared, autoimmune pathology is prevented and chronic levels of infection only rarely result in clinical signs unless the mice become immunocompromised [40].

It is evident that normal modulation virus-specific B cell and antibody responses can increase the severity of acute infection as evidenced by higher viremia at 1 wpi with FV (Fig 6A). Likewise, in humans infected with HIV [41, 42] or hepatitis B virus [43], elevated Tregs correlate with higher viral burdens. However, the counterbalance of preventing autoimmune or immunopathological responses must be of great import to be evolutionarily conserved between mice and man. A high proportion of early B cells display reactivity to self-antigens [44] and maturation of autoreactive B cells and the release of autoantibodies can lead to diseases such as systemic lupus erythematosus or rheumatoid arthritis [45, 46]. In these diseases, reduced Treg frequencies correlate with disease severity [47, 48]. In mice, Treg depletion during vaccination with NP-KLH leads to an outgrowth of primarily antigen-nonspecific B cells [4]. In contrast to our findings, the influenza study demonstrated that Tregs are not always suppressive of antiviral antibody responses [11]. Overall, our results indicate that impaired clearance of certain viruses due to Treg activity could be a consequence of an immune response that is finely tuned to combat pathogens while also limiting immunopathology. Thus, while our findings suggest that Treg depletion could be a promising strategy to enhance the kinetics or magnitude of antibody responses during viral infection, these benefits need to be weighed against the potential for immunopathology or autoimmune disease.

## Supporting information

S1 Fig Treg depletion and gating strategies for lymphocyte populations. (**A-B)** Gating strategy for defined populations with representative FACS plots. All cells were first gated on FSC v. SSC, FSC-A v. FSC-H, and time to select live nucleated single cells. (**B**) B cells were gated as CD4^-^CD19^+^ cells from the live nucleated cell gate. From the B cell gate, GC B cells were selected as CD38^lo^GL7^+^ and plasma cells were selected as CD38^hi^CD138^+^.
(TIF)
