# Supplementary figures and images for: Regulatory T cells suppress virus-specific antibody responses to Friend retrovirus infection
Source: PLoS One. 2018 Apr 3;13(4):e0195402. doi: 10.1371/journal.pone.0195402 (PMC5882174; doi:10.1371/journal.pone.0195402)

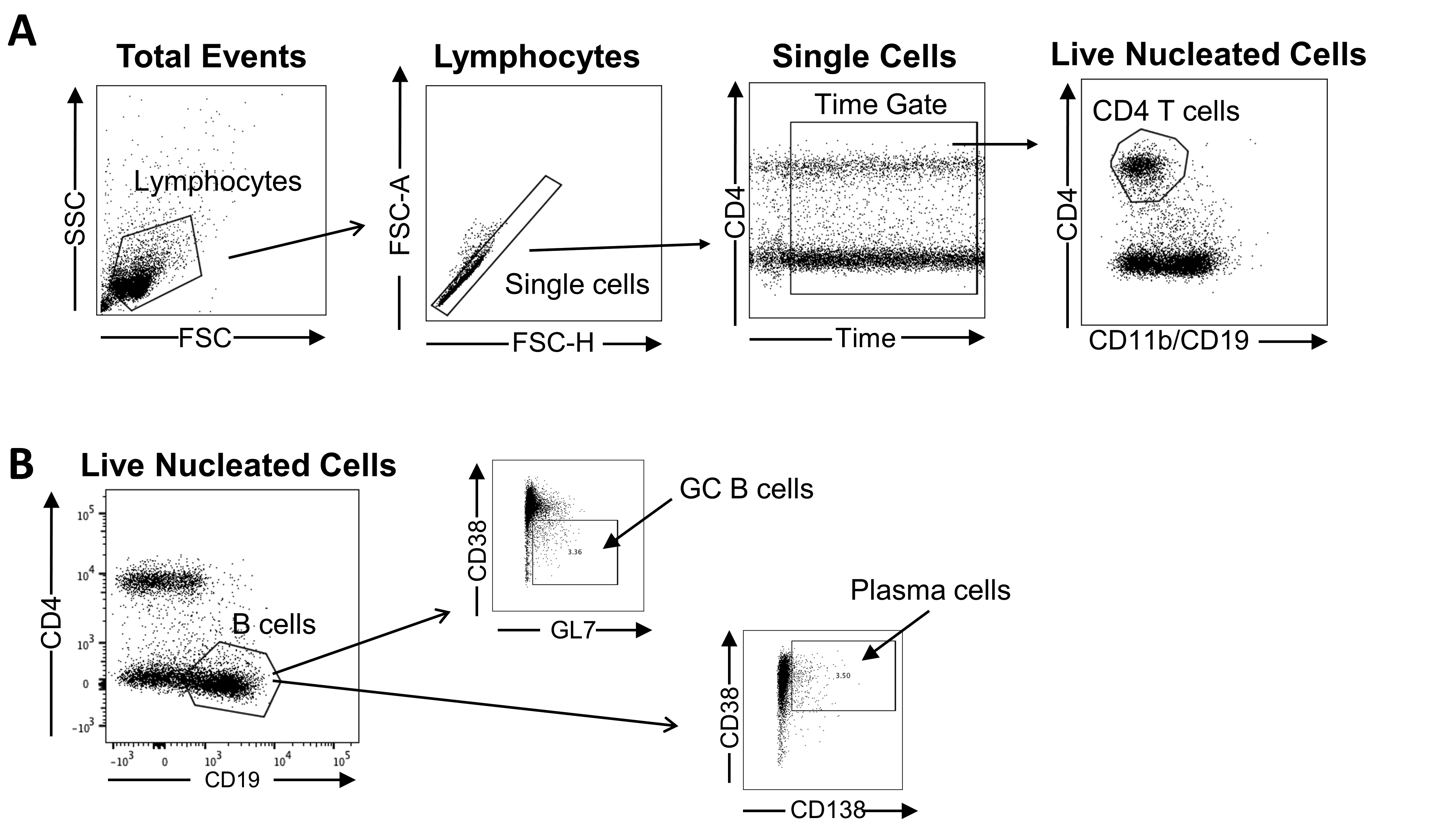

Supplement: S1 Fig — (A-B) Gating strategy for defined populations with representative FACS plots. All cells were first gated on FSC v. SSC, FSC-A v. FSC-H, and time to select live nucleated single cells. (B) B cells were gated as CD4-CD19+ cells from the live nucleated cell gate. From the B cell gate, GC B cells were selected as CD38loGL7+ and plasma cells were selected as CD38hiCD138+. (TIF) [file pone.0195402.s001.TIF]
